# Morphophysiological profiles associated with thermoregulation in dark-coated North Tinerfeña goats and Canária de Pelo sheep in the Canary Islands

**DOI:** 10.1007/s00484-026-03324-z

**Published:** 2026-09-14

**Authors:** Wallace Sostene Tavares da Silva, Robson Mateus Freitas Silveira, Luis Alberto Bermejo, Alexandr Torres Krupij, Débora Andréa Evangelista Façanha

**Affiliations:** 1 Department of Animal Science, Federal Rural University of the Semi-Arid Region (UFERSA), Mossoró, 59625-900 RN Brazil; 2 Department of Animal Science, “Luiz de Queiroz” Agriculture College (ESALQ), University of São Paulo (USP), Piracicaba, 13418-900 São Paulo State Brazil; 3 Departamento de Ingeniería Agraria y del Medio Natural, Escuela Politécnica Superior de Ingeniería – Sección Agraria, Universidad de La Laguna (ULL), Carretera de Geneto nº2, San Cristóbal de La Laguna (Tenerife), 38200 España; 4 Animal Production Unit, Pastures and Forages in Arid and Subtropical Zones, Canarian Institute of Agricultural Research (ICIA), PBOX n° 60, La Laguna, Santa Cruz de Tenerife, 38200 España; 5 University of International Integration of Afro-Brazilian Lusophony (UNILAB), Redenção, 62790-790 CE Brazil

**Keywords:** Thermoregulation, Coat morphology, Phenotypic differentiation, Local breeds

## Abstract

This study evaluated and compared morphophysiological characteristics associated with thermoregulation in North Tinerfeña goats and Canária sheep, both dark-coated and maintained under similar environmental conditions on the island of Tenerife, Spain. A total of 23 adult Canária de Pelo ewes (clinically healthy, multiparous, non-pregnant; mean age 2.5 years; average body weight 39.50 ± 1.25 kg) and 30 North Tinerfeña goats (all females, aged 2–4 years and non-pregnant; average body weight 39.45 ± 3.58 kg) were evaluated. Physiological variables (rectal temperature and respiratory rate), coat morphological traits (coat thickness, hair length, and hair diameter), and environmental variables (air temperature and relative humidity) were measured. Repeated-measures analysis revealed significant population effects for rectal temperature and respiratory rate (*P* < 0.001). Canária sheep exhibited higher rectal temperatures and markedly higher respiratory rates than North Tinerfeña goats, although rectal temperature remained within the physiological range of homeothermy in both populations. North Tinerfeña goats exhibited significantly thicker and longer coats with larger hair diameters than Canária sheep (*P* < 0.001), demonstrating marked population-associated differences in coat morphology. Canonical discriminant analysis performed at the individual-animal level and validated using leave-one-animal-out cross-validation correctly classified 98.1% of animals using physiological traits alone and 100% using either coat morphological traits or the combined set of physiological and morphological variables. The classification tree identified hair length as the primary discriminatory trait between populations, with a threshold of 54.70 mm completely separating the two groups within the evaluated population. These findings demonstrate pronounced population-associated physiological and morphological differentiation related to thermoregulation in dark-coated North Tinerfeña goats and Canária sheep and provide a basis for further investigation of thermoregulatory variation in locally adapted small ruminants.

**Supplementary Information:**

The online version contains supplementary material available at https://doi.org/10.1007/s00484-026-03324-z.

## Introduction

The progressive increase in global temperature and the intensification of extreme climatic events have posed growing challenges to animal production systems, particularly in tropical, subtropical, and insular regions (Silveira et al. 2026). The combination of high air temperatures, intense solar radiation, and increased radiant heat load compromises the thermal balance of animals, with direct impacts on productive performance, reproductive efficiency, and animal welfare (Jongbo et al. 2026). In this context, understanding thermoregulatory responses and phenotypic characteristics associated with thermal adaptation in locally adapted species and breeds is essential for the development of resilient livestock production systems under climate change scenarios (Silveira et al. 2024).

Among domestic ruminants, goats and sheep play a strategic role in environmentally restrictive regions, being commonly raised in arid, semi-arid, and rugged landscapes. Despite occupying similar production niches, these species exhibit marked differences in physiology, behavior, and morphology, resulting in distinct thermoregulatory patterns (Sejian et al. 2018). Consistent evidence indicates that goats tend to be more heat tolerant than sheep, reflecting a long history of natural and anthropogenic selection in warmer and drier environments (Aboul-Naga et al. 2021).

Thermoregulation in small ruminants results from the interaction between physiological mechanisms—such as respiratory rate and body temperature—and morphological attributes, particularly coat characteristics (da Silva, 2025ab). Respiratory rate is recognized as one of the most sensitive indicators of heat stress, providing an early reflection of increased evaporative heat dissipation (Berihulay et al. 2019; Façanha et al. 2020). However, intense and prolonged physiological responses entail higher energetic costs and may compromise productive efficiency (Lima et al. 2022). In this context, coat traits such as thickness, hair length, and fiber diameter play a central role in modulating thermal balance by influencing solar radiation absorption, thermal insulation, and the efficiency of sensible heat exchange with the environment (McManus et al. 2020).

Local breeds represent particularly relevant biological models for studying thermoregulatory variation under different environmental conditions (Senczuk et al. 2024; Silveira et al. 2026). In the Canary Islands, North Tinerfeña goats and Canária sheep, both dark-coated, have evolved over centuries in an environment characterized by high solar radiation, seasonal thermal variability, volcanic terrain, and water limitations (Silveira et al. 2024; Façanha et al. 2023). These environmental conditions are associated with distinct physiological and morphological phenotypic characteristics. Despite their zootechnical, cultural, and ecological importance, systematic studies investigating the thermoregulatory responses and associated morphological traits of these local breeds—particularly under natural exposure conditions—remain scarce.

Furthermore, to date, no studies have simultaneously and comparatively evaluated two small ruminant species—dark-coated goats and sheep— raised under similar environmental conditions in the Canary Islands, integrating physiological responses and coat traits using multivariate approaches. This gap limits the understanding of the distinct morphophysiological patterns exhibited by closely related yet biologically distinct species when facing similar thermal challenges.

Based on previous evidence indicating that goats and sheep differ in physiological and morphological responses to thermal challenges, we hypothesized that North Tinerfeña goats and Canária sheep, despite sharing dark coats and being exposed to similar environmental conditions in the Canary Islands, would exhibit distinct morphophysiological patterns associated with thermoregulation. We further hypothesized that the integration of physiological and coat-related traits would allow the identification of distinct population-associated morphophysiological profiles related to thermoregulation.

Within this framework, the objective of the present study was to evaluate and compare the physiological responses and coat morphological traits of dark-coated North Tinerfeña goats and Canária sheep in the Canary Islands. By integrating a multimodel approach, this study aimed to characterize population-associated morphophysiological patterns related to thermoregulation and to improve the understanding of physiological and morphological variation in small ruminants exposed to environments with high radiant heat load.

## Materials and methods

### Location and environmental conditions

This study was conducted on the island of Tenerife, Canary Islands, Spain. Experimental conditions were designed to ensure comparable housing, management, and feeding systems between goats and sheep. All measurements were performed under the same climatic classification (Mediterranean climate with hot and dry summers – Csa, Köppen–Geiger), characterized by relatively stable air temperatures and moderate thermal amplitude throughout the year. The island presents predominantly mountainous relief with volcanic soils typical of insular environments.

### Animals and production systems

A total of 23 adult Canária de Pelo ewes and 30 North Tinerfeña goats were evaluated. All animals were clinically healthy, multiparous, non-pregnant females aged between 2 and 4 years. Mean body weight was 39.50 ± 1.25 kg for the sheep and 39.45 ± 3.58 kg for the goats.

Both populations were maintained under the same intensive production system at the Island Center for Conservation and Improvement of Animal Genetic Resources (ICIA), Tenerife, Spain. Animals were housed in facilities roofed with metal sheets, laterally open on the longest side (4 m), with concrete flooring. Concentrate feed based on corn and soybean meal was supplied during the early morning hours (06:00 h), whereas chopped forage composed of native plant species was offered throughout the day. Diets met the nutritional requirements recommended by the NRC (2007) for small ruminants. Water was available ad libitum, and all animals followed a routine sanitary program in accordance with regional veterinary guidelines.

### Data collection

Data were collected from the same animals throughout the entire 12-month experimental period, ensuring exposure to the same seasonal environmental conditions and reducing potential confounding effects associated with management or climate. The evaluated animals belonged to a restricted age range and consisted exclusively of adult, non-pregnant females, minimizing potential age- and parity-related effects on physiological and coat traits.

All animals were maintained at the Island Center for Conservation and Improvement of Animal Genetic Resources (ICIA, Spain), Valle de Guerra, Tenerife, Spain (28°31′ N, 16°22′ W; approximately 300 m above sea level). Goats and sheep were managed under the same husbandry protocols, including feeding, housing, health care, and reproductive management.

Measurements were performed once per month throughout the 12-month experimental period. Physiological and coat morphological traits were assessed during each monthly sampling session following standardized procedures. Prior to each data collection session, animals were kept in a single holding pen for 30 min to minimize the influence of handling and acclimation stress on physiological responses. All variables were measured following a predetermined sequence established before the beginning of the study. Thermoregulatory variables were assessed first, followed by morphological measurements, in order to minimize potential interference among measurements and reduce the influence of handling on the recorded responses. This procedure remained consistent throughout the experimental period to ensure standardization of data collection. Meteorological variables were recorded simultaneously with the thermoregulatory variables, ensuring paired environmental and physiological data for each animal.

### Thermoregulatory responses

Respiratory rate (RR) was measured by direct auscultation over a 1-min interval by trained personnel following a standardized protocol. Rectal temperature (RT) was recorded using a digital clinical thermometer (accuracy ± 0.1 °C; Omron Flex Temp digital thermometer, China) inserted approximately 5 cm into the rectum, maintaining contact with the rectal mucosa. All measurements were performed using the same procedures throughout the study period to minimize observer-related variation.

### Coat morphological traits

Samples were collected from the left flank region, a standardized anatomical site commonly used in studies of coat characteristics due to its representativeness of the overall coat profile. Approximately 10–20 hairs were manually removed using duckbill pliers, ensuring that the fibers were collected as close as possible to the skin surface while avoiding damage to the surrounding coat. All collections were performed following the same protocol throughout the study period to ensure consistency among animals and sampling occasions.

Coat thickness was measured in situ at the same anatomical site using a millimetric metal ruler equipped with a slider, inserted perpendicularly through the coat until contacting the skin surface. The slider was then adjusted to the outermost layer of the coat, and the distance was recorded.

Hair length was determined in the laboratory using a digital caliper (Digimess, model 100.174BL). Following the methodology proposed by Udo (1978), the 10 longest hairs within each sample were measured, and their arithmetic mean was used to represent coat length. This procedure is widely employed in studies of animal coat characteristics because the longest fibers are considered the main contributors to the thermal insulation properties of the coat. Hair diameter was measured on the same fibers using a digital micrometer (Mitutoyo), and the arithmetic mean was subsequently calculated (Lee 1953).

### Environmental variables

Air temperature and relative humidity were recorded using a digital thermo-hygrometer (TGD-400, Instrutherm, São Paulo, Brazil) and black globe thermometer in the same setting and time as the morphophysiological measurements, yielding paired ambient data per animal. From these readings, the Radiant Heat Load (RHL) was calculated according to Silva (2008):

$$\mathrm{RHL}\;=\;1.053\;\times\;\mathrm{hc}\;(\mathrm{TGN}\;-\;\mathrm{Ta})\;+\;\sigma\mathrm{TGN}^4(\mathrm{W/m}^2)$$

where: hc = convective heat transfer coefficient (W/m²·K); TGN = black globe temperature (K); Ta = air temperature (K); σ = Stefan–Boltzmann constant (5.6697 × 10⁻⁸ W/m²·K⁴).

This approach is widely adopted in studies evaluating thermal comfort and adaptation of livestock under field conditions, facilitating comparison with previous research.

### Statistical analysis

The data were initially assessed for the assumptions underlying the statistical models. Normality was evaluated based on the distribution of model residuals, while homogeneity of variances between groups was also assessed prior to inferential analyses.

The physiological variables, rectal temperature (RT) and respiratory rate (RR), and the coat morphological traits, coat thickness (CT), hair length (HL), and hair diameter (HD), were analyzed using repeated-measures analysis of variance. Each animal was evaluated once per month throughout the 12-month experimental period, resulting in 12 repeated monthly observations per animal. Population was considered the between-subject factor, month as the within-subject factor, and each animal as the experimental subject. The effects of population, month, and the population × month interaction were evaluated for each trait. When the assumption of sphericity was not met, degrees of freedom were adjusted using the Greenhouse–Geisser correction.

Month was included in the model to account for temporal variation throughout the longitudinal study rather than to test seasonality as a primary hypothesis. The main effect of interest was populations, since the primary objective was to compare the morphophysiological profiles of dark-coated North Tinerfeña goats and Canária sheep. Because the populations factor comprised only two levels, the comparison between populations was performed directly through the main effect of populations, without the need for post hoc multiple-comparison tests.

Additionally, a statistical power sensitivity analysis was conducted considering the 53 animals included in the study, comprising 30 North Tinerfeña goats and 23 Canária sheep, a two-sided significance level of α = 0.05, and statistical power of 80%. The analysis indicated that the available sample size was capable of detecting standardized between-group differences of approximately Cohen’s d ≥ 0.79, corresponding to large effect sizes. Furthermore, significant differences between populations were detected for all morphophysiological traits evaluated, supporting the adequacy of the sample size for the primary comparative objective of the study. Nevertheless, the ability to detect effects of smaller magnitude would be more limited. Statistical significance was established at *P* < 0.05 for all analyses.

To explore the main multivariate patterns and relationships among physiological, morphological, and environmental variables, an exploratory factor analysis (EFA) was conducted using standardized (z-score) physiological, morphological, and environmental variables. Factor extraction was followed by varimax orthogonal rotation to improve interpretability. Sampling adequacy and factorability of the data were verified using the Kaiser–Meyer–Olkin (KMO) measure and Bartlett’s test of sphericity, respectively. Factor loadings and biplots were used to interpret the relationships among variables and to visualize populations separation in multivariate space.

For the multivariate classification analyses, repeated observations were summarized at the individual-animal level using the mean value of each trait, so that each animal represented a single independent case. Canonical discriminant analysis was performed separately using physiological variables (RT and RR), coat morphological traits (CT, HL, and HD), and the combined set of physiological and morphological traits. Because only two populations were compared, a single canonical discriminant function was estimated for each model. The significance of the discriminant function was assessed using Wilks’ Lambda, and the relative contribution of each variable to populations discrimination was evaluated using standardized canonical discriminant function coefficients. Classification performance was internally validated using leave-one-animal-out cross-validation (LOOCV), in which each animal was sequentially excluded from model fitting and subsequently classified using the discriminant function derived from the remaining individuals. Overall cross-validated accuracy, population-specific accuracy, and balanced accuracy were calculated.

Finally, a classification tree analysis was performed at the individual-animal level to identify the traits that most effectively discriminated North Tinerfeña goats from Canária sheep and to determine interpretable threshold values associated with population differentiation. Model performance was assessed using animal-level cross-validation, ensuring that each animal used for validation was not simultaneously included in model training. This approach was adopted to reduce the risk of overfitting and to provide a more robust estimate of the discriminatory performance of the classification tree. Statistical analysis was performed using IBM SPSS Statistics for Windows, Version 25.0 (IBM Corp., Armonk, NY, USA).

## Results

Rectal temperature differed between populations, although values remained within the physiological range of approximately 39–40 °C for both (Reece et al. 2015; Table 1). Canária sheep exhibited slightly higher rectal temperatures, whereas North Tinerfeña goats showed lower values (*P* < 0.001). Respiratory rate was substantially higher in Canária sheep (83.47 ± 1.10 breaths min⁻¹; *P* < 0.001), nearly double that observed in North Tinerfeña goats (39.33 ± 1.40 breaths min⁻¹).

**Table 1:** Physiological responses and coat morphological traits of North Tinerfeña goats and Canária sheep

Trait	North Tinerfeña	Canária	*P* population
RT (°C)	39.18 ± 0.04	39.68 ± 0.03	< 0.001
RR (breaths/min)	39.33 ± 1.40	83.47 ± 1.10	< 0.001
CT (mm)	6.84 ± 0.39	0.80 ± 0.01	< 0.001
HL (mm)	127.63 ± 4.06	21.28 ± 0.25	< 0.001
HD (mm)	0.074 ± 0.001	0.026 ± 0.0004	< 0.001

Values are presented as mean ± SEM. P population represents the P-value for the main effect of populations obtained from the repeated-measures analysis. RT = rectal temperature (°C); RR = respiratory rate (breaths/min); CT = coat thickness (mm); HL = hair length (mm); HD = hair diameter (mm); SEM = standard error of the mean. Statistical significance was established at *P* < 0.05

Coat traits revealed contrasting morphological patterns between the two evaluated populations (Table 1). North Tinerfeña goats exhibited a much thicker coat (6.84 ± 0.39 mm), longer hair (127.63 ± 4.06 mm), and larger hair diameter (0.074 ± 0.001 mm**)** compared with Canária sheep. In contrast, Canária sheep showed markedly thinner (0.80 ± 0.01 mm) and shorter coats (21.28 ± 0.25 mm), with a smaller hair diameter (0.026 ± 0.0004 mm). All coat morphological traits differed significantly between populations (*P* < 0.001; Table 1).

In addition to the main effect of population, month significantly affected all physiological and morphological traits evaluated (*P* < 0.001 for all). Significant population × month interactions were also observed for all traits (*P* < 0.001 for all), indicating that the magnitude of the physiological and morphological differences between North Tinerfeña goats and Canária sheep varied across months. These temporal effects were accounted for within the repeated-measures framework, while the primary focus of the study remained the comparison between populations.

Monthly patterns are presented in Supplementary Figure S1. The magnitude of the differences between the two evaluated populations varied throughout the sampling period. Canária de Pelo sheep generally exhibited higher rectal temperatures and respiratory rates, although these differences narrowed during some months, particularly toward the end of the sampling period. Differences in coat morphology persisted throughout the study, but their magnitude also varied over time. The difference in coat thickness decreased markedly after month 6, whereas the difference in hair length increased. The difference in hair diameter became smaller during months 10–12 because of the increase observed in Canária de Pelo sheep.

Discriminant analysis confirmed a strong differentiation between the two evaluated populations when each animal was considered as an independent classification unit (Table 2). Using only physiological indicators, leave-one-animal-out cross-validation correctly classified 98.1% of the animals, corresponding to 96.7% of North Tinerfeña goats and 100% of Canária sheep. RR showed a greater contribution to the discriminant function than rectal temperature.

**Table 2:** Canonical discriminant analysis of physiological and morphological profiles of North Tinerfeña goats and Canária sheep and classification performance based on leave-one-animal-out cross-validation

Indicators	LOOCV Accuracy (%)	North Tinerfeña (%)	Canária (%)	Variance Explained by F1 (%)	Wilks’ λ	*P*-value	Canonical Correlation	Balanced Accuracy (%)	Main Traits¹
Physiological	98.1	96.7	100.0	100	0.080	< 0.001	0.959	98.3	RR (1.124) > RT (0.254)
Morphological	100.0	100.0	100.0	100	0.038	< 0.001	0.981	100.0	HD (0.809) > HL (0.550) > CT (0.158)
All traits	100.0	100.0	100.0	100	0.015	< 0.001	0.992	100.0	RR (0.898) > HD (0.703) > HL (0.632) > CT (0.037) > RT (0.032)

¹ Main traits are ranked according to the absolute magnitude of the standardized canonical discriminant function coefficients. Larger absolute coefficients indicate a greater relative contribution to discrimination between populations. The sign of the coefficient represents the direction of association with the discriminant function and not its relative importance

Abbreviations: *RT* rectal temperature, *RR* respiratory rate, *CT* coat thickness, *HL* hair length, *HD* hair diameter, *F*1 first canonical discriminant function, *LOOCV* leave-one-animal-out cross-validation

When only coat morphological traits were considered, leave-one-animal-out cross-validation resulted in 100% correct classification of both populations. Hair diameter showed the largest standardized canonical discriminant coefficient, followed by hair length and coat thickness. The morphological discriminant function was highly significant (Wilks’ λ = 0.038; *P* < 0.001), with a canonical correlation of 0.981.

Combining physiological and morphological traits also resulted in 100% cross-validated classification accuracy. Because only two populations were compared, a single canonical discriminant function was obtained, explaining 100% of the discriminant variance. This function showed a very strong association with population differentiation (Wilks’ λ = 0.015; *P* < 0.001; canonical correlation = 0.992). In the combined model, respiratory rate, hair diameter, and hair length showed the greatest relative contributions to discrimination, followed by coat thickness and rectal temperature.

**Fig. 1:**
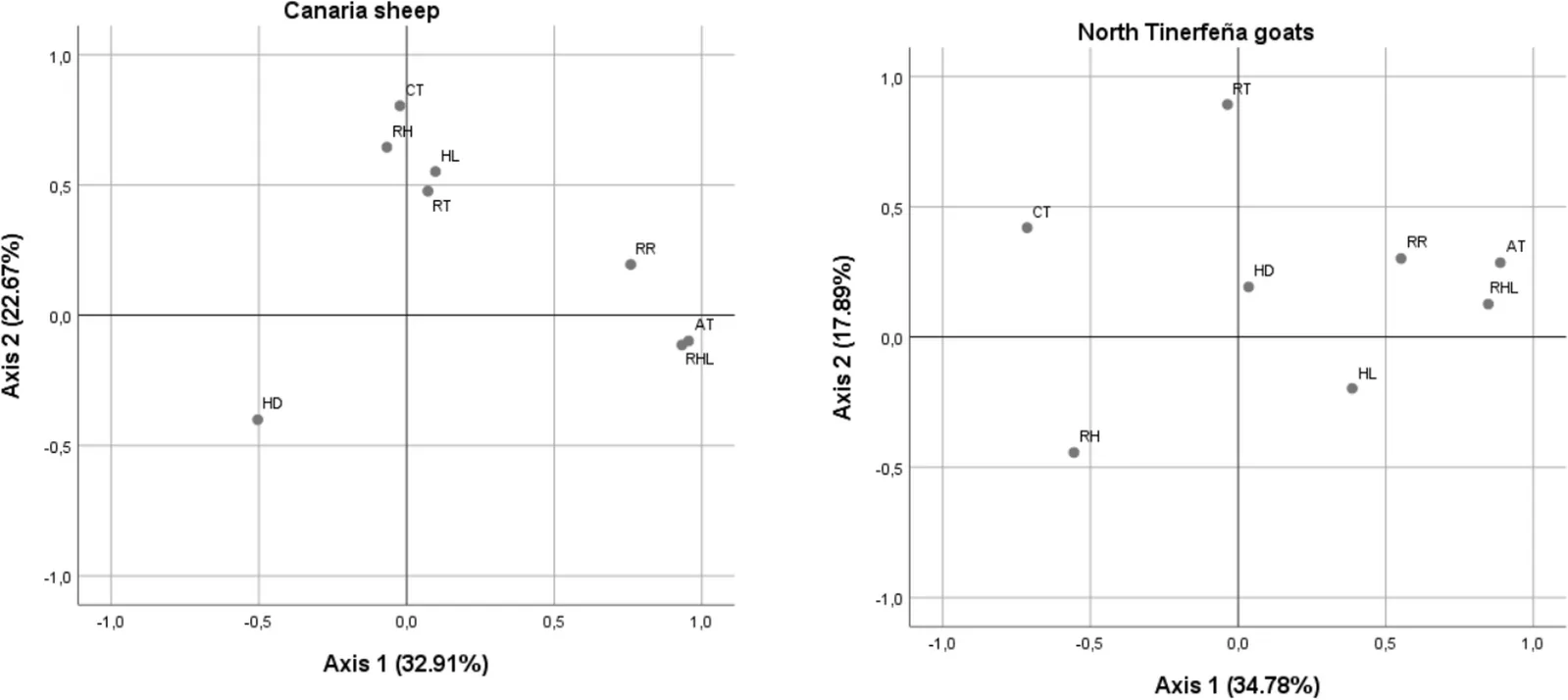
Exploratory Factor Analysis (EFA) biplots by goat and sheep breed (North Tinerfeña and Canaria) showing the contribution and association among physiological indicators, coat traits, and environmental variables. The percentages on the axes correspond to the variance explained by each component. RT - rectal temperature (°C); RR - respiratory rate (breaths/min); CT - coat thickness (mm); HL - hair length (mm); HD - hair diameter (mm), AT - air temperature (°C), RH – relative humidity (%), RHL, -radiant heat load (w m²)

Exploratory factor analysis (EFA) biplots (Fig. 1) revealed population-specific patterns. In Canária sheep, coat thickness, relative humidity, and hair length were strongly associated with the second axis, while hair diameter loaded in the opposite direction. In North Tinerfeña goats, rectal temperature, coat thickness, respiratory rate, and hair diameter loaded positively, whereas relative humidity contributed negatively. In both populations, respiratory rate was positively associated with radiant heat load.

**Fig. 2:**
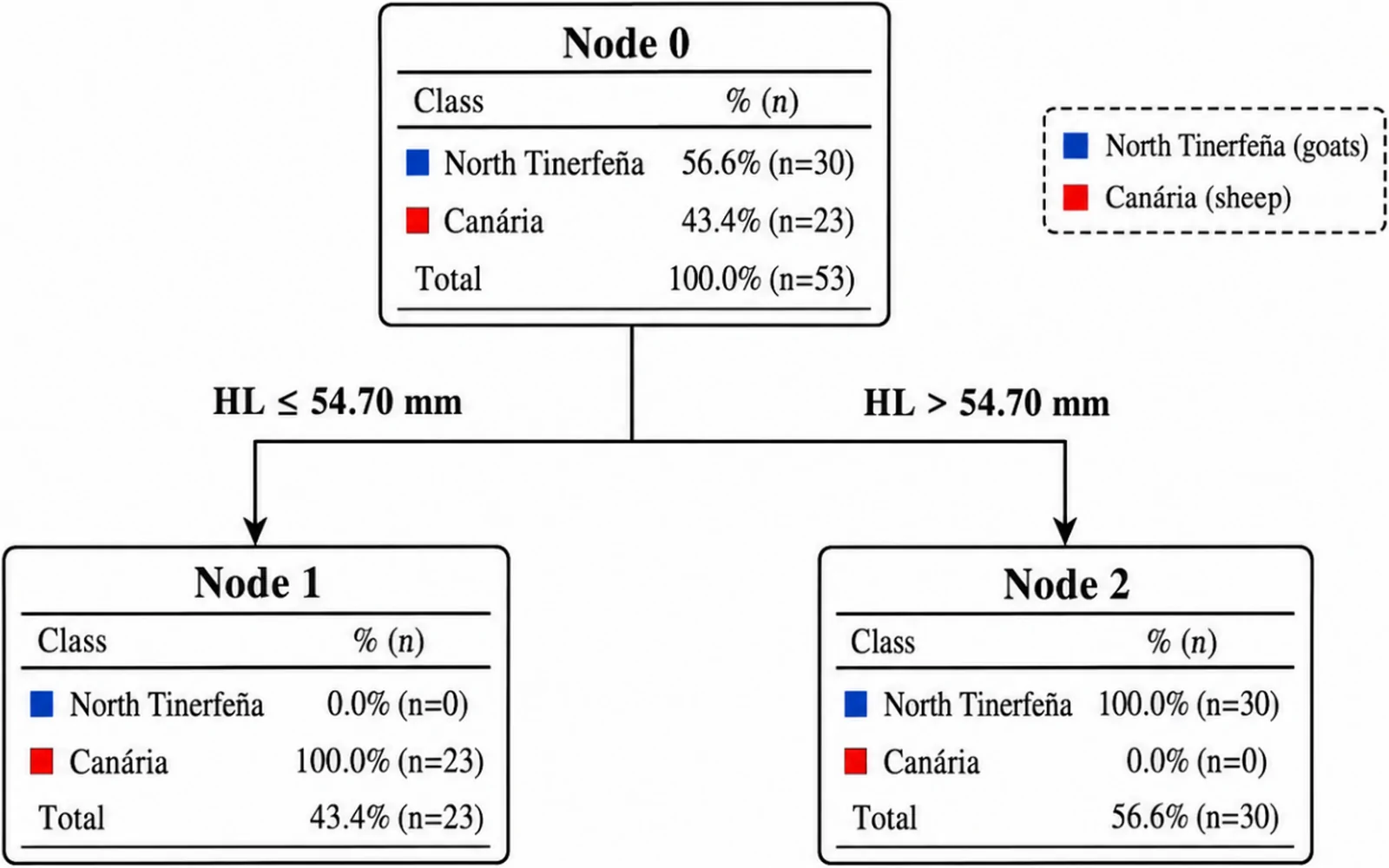
Classification tree based on hair length (HL) used to discriminate between North Tinerfeña goats and Canária sheep

The classification tree developed at the individual-animal level identified hair length as the single discriminatory variable (Fig. 2). A threshold of 54.70 mm completely separated the two populations: animals with mean hair length ≤ 54.70 mm were classified as Canária sheep, whereas those with mean hair length > 54.70 mm were classified as North Tinerfeña goats. Classification accuracy remained 100% under animal-level cross-validation. These findings identify hair length as a strong phenotypic marker of population differentiation within the evaluated population, rather than as direct evidence of an adaptive mechanism.

## Discussion

The results of this study indicate that, although dark-coated goats and sheep were exposed to similar environmental conditions, their physiological and morphological profiles differed between populations. Canária sheep exhibited higher respiratory rates than North Tinerfeña goats, suggesting a greater reliance on respiratory heat dissipation. However, this difference should be interpreted cautiously, as respiratory rate may also be influenced by population-specific physiological and metabolic characteristics. Likewise, although Canária de Pelo sheep showed slightly higher rectal temperatures, the magnitude of these differences was relatively small, indicating that both populations were able to maintain thermal homeostasis under the environmental conditions evaluated. These findings suggest associations between populations and distinct thermoregulatory patterns rather than demonstrating differences in heat sensitivity or adaptive capacity.

Studies indicate that goats tend to be more heat-resilient than sheep due to greater plasticity in physiological and morphological responses and a long history of selection in arid environments (Aboul-Naga et al. 2021; Silveira et al. 2026). In this context, the lower rectal temperatures observed in North Tinerfeña goats compared with Canária sheep are consistent with previous reports of physiological differences between locally adapted goat and sheep populations exposed to thermal challenges (Nair et al. 2021). However, the present comparison does not allow these differences to be attributed directly to greater adaptive capacity in North Tinerfeña goats.

The marked difference in respiratory rate between the two evaluated populations supports the existence of distinct physiological responses. In the present study, Canária de Pelo sheep exhibited nearly double the respiratory rate observed in North Tinerfeña goats. Respiratory rate is recognized as a more sensitive indicator of heat stress than rectal temperature in small ruminants, reflecting early activation of evaporative heat loss (Maia et al. 2016; Lima et al. 2022). Studies on goats exposed to high temperatures report that, although respiratory rate increases, these animals rapidly restore homeothermy by adjusting sweating and acid–base balance (Façanha et al. 2020; Ferreira et al. 2020). In contrast, sheep exposed to high temperatures and solar radiation often show persistent elevations in respiratory rate and rectal temperature, indicating greater physiological effort to dissipate heat (Pulido-Rodríguez et al. 2021). Thus, under the evaluated conditions, the lower respiratory rates observed in North Tinerfeña goats may be associated with their morphophysiological characteristics, whereas the higher respiratory rates observed in Canária de Pelo sheep may indicate a greater contribution of respiratory evaporative heat dissipation.

Differences between the two evaluated populations were even more evident when coat traits were considered. The thicker, longer coats with larger fiber diameter observed in North Tinerfeña goats contrast sharply with the short and thin coats of Canária sheep. Studies on hair sheep breeds (e.g., Morada Nova, Santa Inês, West African Dwarf) demonstrate that finer, shorter, and thinner coats favor sensible heat dissipation, resulting in lower rectal temperature and respiratory rate under heat stress (Okourwa 2015; Costa et al. 2018; Mascarenhas et al. 2023). Similarly, studies in goats indicate that excessively dense or thick coats can influence heat exchange and increase reliance on evaporative mechanisms, whereas coats with lower thickness and density but moderate length may contribute to the balance between solar protection and heat exchange with the environment (da Silva et al. 2025a, b).

In the present study, North Tinerfeña goats exhibited a thicker and longer coat than Canária sheep. Such coat characteristics have been described in goat populations raised under environments with high solar radiation or altitude and may influence the transfer of radiant heat toward the skin. However, the present study did not directly quantify heat transfer through the coat, and this interpretation should therefore be considered a plausible association rather than a demonstrated mechanism. Studies on Canary Island goat populations show seasonal variation in coat surface temperature, suggesting that coat characteristics may participate in the interaction between the animal and changes in radiation and ambient temperature (Silveira et al. 2024; da Silva et al. 2025a, b). Conversely, Canária sheep displayed short and thin coats, similar to patterns described for Brazilian hair sheep exposed to semi-arid conditions, which may be associated with reduced insulation and greater potential for sensible heat exchange (Façanha et al. 2023). In dark-coated animals, shorter hair and reduced fiber diameter have also been associated with differences in thermal exchange; however, the present data do not demonstrate that these characteristics constitute adaptive mechanisms per se (McManus et al. 2020).

The multivariate analyses reinforce the existence of pronounced physiological and morphological differentiation between North Tinerfeña goats and Canária sheep. The inclusion of coat traits revealed markedly distinct phenotypic profiles between the two populations, highlighting the importance of coat morphology in characterizing these differences. These findings reinforce that the two evaluated populations exhibited distinct combinations of physiological and morphological characteristics associated with thermoregulation. Nevertheless, these differences should be interpreted as population-associated phenotypic patterns rather than as direct evidence of adaptive mechanisms or superior heat tolerance.

The contribution of both physiological and coat-related traits to population differentiation highlights the multifactorial nature of thermoregulation in small ruminants. RR and coat morphology represent complementary components of the interaction between animals and their thermal environment (da Silva et al. 2026a). In particular, differences in hair diameter and length may influence insulation and heat exchange at the body surface, whereas respiratory rate reflects the physiological component of heat dissipation. A similar integrative pattern has been reported in locally adapted Brazilian goats, in which physiological responses and coat characteristics varied jointly under semiarid conditions, and coat thickness emerged as an important trait for differentiating phenotypic profiles (da Silva et al. 2026b). The combined importance of these traits suggests that the contrasting profiles observed between North Tinerfeña goats and Canária sheep arise from distinct combinations of physiological and morphological characteristics rather than from a single thermoregulatory attribute.

The classification tree provided a complementary and highly interpretable result. Hair length emerged as the single splitting variable, with a threshold of 54.70 mm completely separating the two populations in the present population. Animals with mean hair length ≤ 54.70 mm were classified as Canária sheep, whereas those with values > 54.70 mm were classified as North Tinerfeña goats. Importantly, classification accuracy remained 100% under animal-level cross-validation. Thus, hair length can be regarded as a strong phenotypic descriptor of population differentiation in the evaluated population, rather than as direct evidence of a specific adaptive mechanism.

From a practical perspective, hair length and respiratory rate emerged as useful phenotypic descriptors of the distinct thermoregulatory profiles observed in North Tinerfeña goats and Canária sheep under the evaluated environmental conditions. These traits may therefore be useful for phenotypic characterization and field monitoring of dark-coated small ruminants. However, their application as selection criteria or management indicators requires further validation using direct measures of heat tolerance, productive performance, and genetic parameters.

Because complete discrimination was retained after leave-one-animal-out cross-validation, the high classification performance is consistent with the pronounced phenotypic differentiation between North Tinerfeña goats and Canária sheep, particularly in coat-related traits. Nevertheless, because the analysis was based on a single experimental population, validation in independent populations would be valuable to determine the broader applicability of these discriminatory patterns.

The significant population × month interactions indicate that the magnitude of the differences between North Tinerfeña goats and Canária de Pelo sheep was not constant throughout the sampling period. The temporal variation in rectal temperature and respiratory rate may reflect differences in how the two populations adjusted their physiological responses to changing environmental conditions. Although Canária de Pelo sheep generally maintained higher values, the narrowing of these differences in some months suggests that the contrast between populations depended partly on the sampling period. Temporal changes were also evident in coat traits, particularly the marked reduction in the difference in coat thickness after month 6 and the increased difference in hair length. These patterns may reflect population-specific dynamics of coat growth, renewal, or shedding (Façanha et al. 2023). Similarly, the reduced difference in hair diameter during months 10–12 resulted from an increase in Canária de Pelo sheep. However, because coat renewal and related mechanisms were not directly evaluated, these temporal patterns should be interpreted cautiously. Overall, the results demonstrate that the observed differences between the two evaluated populations were dynamic rather than uniform throughout the year.

## Limitations

The study presents some limitations that should be considered when interpreting the findings. First, only one breed from each species was evaluated—North Tinerfeña goats and Canária de Pelo sheep. Consequently, species and breed effects are completely confounded, and the observed differences cannot be attributed uniquely to either taxonomic level. The findings should therefore be interpreted as differences between these two evaluated populations and should not be generalized to goats and sheep as species.

Second, pedigree information and genomic data were not available, limiting inferences regarding the genetic basis and heritability of the evaluated traits. Third, direct mechanistic measurements, such as sweating rate, skin blood flow, metabolic heat production, and acid–base responses, were not obtained. Therefore, the physiological and morphological patterns identified in this study should be interpreted as traits associated with thermoregulation rather than as direct demonstrations of specific thermoregulatory mechanisms.

Finally, although the discriminatory performance of the multivariate models was assessed using internal cross-validation at the animal level, independent external populations were not available for validation. Future studies including additional dark-coated breeds and independent populations may help determine the broader generalizability and applicability of the morphophysiological patterns identified in the present study.

## Conclusion

Dark-coated North Tinerfeña goats and Canária sheep exhibited distinct physiological and morphological profiles associated with thermoregulation under the evaluated environmental conditions. Canária sheep were characterized by higher respiratory rates and shorter, thinner coats with smaller hair diameter, whereas North Tinerfeña goats exhibited lower respiratory rates and thicker, longer coats with larger hair diameter. These findings demonstrate marked population-associated morphophysiological differentiation between the two populations.

Multivariate analyses further highlighted the importance of coat morphology in distinguishing the populations. Hair diameter and hair length were among the traits contributing most strongly to morphological differentiation, while hair length emerged as the primary discriminatory variable in the classification tree. The complete cross-validated separation obtained from morphological traits indicates pronounced phenotypic differentiation within the evaluated population.

Overall, the present study contributes to the characterization of thermoregulation-related morphophysiological variation in dark-coated small ruminants from the Canary Islands. These findings should be interpreted as evidence of population-associated phenotypic differences rather than direct demonstration of adaptive mechanisms. Further studies involving additional dark-coated breeds, direct measurements of heat exchange, genetic information, and independent populations are needed to determine the broader biological and practical relevance of these traits. 

## Supplementary Information

Below is the link to the electronic supplementary material.

Supplementary Material 1

## Data Availability

Data are available from the corresponding author upon reasonable request.
