# Supplementary material for: Morphophysiological profiles associated with thermoregulation in dark-coated North Tinerfeña goats and Canária de Pelo sheep in the Canary Islands
Source: Int J Biometeorol. 2026 Sep 14;70(9):252. doi: 10.1007/s00484-026-03324-z (PMC13574850; doi:10.1007/s00484-026-03324-z)
Supplement: Supplementary file 1 [file 484_2026_3324_MOESM1_ESM.docx]

**Figure S1.** Monthly patterns of physiological and coat morphological traits in North Tinerfeña goats and Canária de Pelo sheep. Monthly means and standard errors of the mean are presented for (A) rectal temperature, (B) respiratory rate, (C) coat thickness, (D) hair length, and (E) hair diameter.

*Note:* North Tinerfeña goats (n = 30) and Canária de Pelo sheep (n = 23) were evaluated across 12 consecutive sampling months.
